# Non-pharmacological treatment of anxiety in general practice: a scoping review

**DOI:** 10.1080/02813432.2026.2645713

**Published:** 2026-03-23

**Authors:** Eva Rix, Laura Felding, Sophia Ingeborg Vang, Gritt Overbeck

**Affiliations:** Department of Public Health, The Research Unit for General Practice, University of Copenhagen, Copenhagen, Denmark

**Keywords:** Anxiety disorders, general practice, digital health, health services implementation, non-pharmacological

## Abstract

**Background:**

Anxiety is a common problem amongst the population, including patients in general practice. Treatment often relies on medication, but non-pharmacological alternatives may offer safer or more sustainable options, particularly for mild to moderate anxiety. There is a limited overview of which non-pharmacological treatments have been studied or implemented in general practice.

**Objectives:**

This scoping review aims to explore and describe existing evidence on non-pharmacological interventions for anxiety in general practice, providing an overview of available treatment approaches and identifying gaps to inform future research.

**Methods:**

A scoping review was made following the PRISMA Extension for Scoping Reviews (PRISMA-ScR) guidelines. Data was collected using keywords in several databases (PubMed, CINAHL and APA PsycInfo) and selected based on predefined eligibility criteria.

**Results:**

Three thousand four hundred and twenty-five articles were screened and 64 were assessed. A total of seven peer-reviewed articles were included. Three themes emerged among the interventions. These were digital interventions, step-wise and low-intensity care models, and body-based interventions. The interventions were delivered through various formats, including mobile apps, online platforms and in-person clinics. The studies all targeted patients with mild to moderate anxiety. Despite differences in methodology and populations, the interventions showed a positive effect.

**Conclusions:**

This scoping review highlights that while the evidence is still limited, the non-pharmacological interventions mentioned in this review have shown promise in the treatment of anxiety. Further research is needed to assess the effectiveness, implementation and real-world impact of these approaches in general practice.

## Introduction

Anxiety disorders are among the most prevalent mental health issues globally and represent a significant challenge for healthcare systems. Anxiety disorders affect an estimated 5.4% of the total EU population and 4% worldwide [1,2]. In a lifetime, 14% of the European population will experience an anxiety disorder [3,4].

Anxiety disorders, as classified in the ICD-10, encompass a range of mental health conditions characterized by excessive fear, worry and associated behavioral disturbances [5]. Patients may present with both psychological symptoms, such as tension, nervousness and catastrophic thinking, as well as physical symptoms, including muscle tension, restlessness, fatigue, tachycardia, dizziness, sweating, headaches and gastrointestinal distress [6].

General practitioners (GPs) are often the first point of contact for patients presenting with anxiety, placing them in a central position to initiate early intervention or refer to specialist services when needed [7,8]. Given the, often considerable, waiting times for mental health care treatment, managing anxiety disorders within general practice is not only appropriate but also a pragmatic necessity [9,10]. GPs frequently have established, long-term professional relationships with their patients and possess in-depth knowledge of their medical history, life circumstances and prior health challenges [11]. This continuity enables the formation of a therapeutic alliance that fosters trust, facilitates communication and enhances patient engagement. All critical elements in the effective treatment of anxiety [12]. Because this alliance is typically already in place, valuable time is saved, allowing for earlier intervention in both diagnosis and treatment compared to referral to a specialist within mental health care [13,14]. Importantly, without appropriate intervention, anxiety can lead to worsening symptoms, decreased quality of life, and chronification, making early treatment essential to prevent the escalation of the disorder. Chronification refers to the transition from acute or episodic anxiety to a persistent condition, often linked to prolonged distress, higher healthcare use and reduced daily functioning [4,15]. Even though pharmacological treatment, like benzodiazepines and antidepressants, is often the first choice, there are known limitations of using pharmacological treatment, such as side effects and lack of effect [16]. There is also a growing patient preference for non-pharmacological alternatives [17]. These include psychological interventions such as cognitive behavioral therapy (CBT) and acceptance and commitment therapy (ACT), digital treatment formats like internet-based therapy, lifestyle interventions like physical activity, and body-based approaches like acupuncture [18].

Anxiety disorders are highly prevalent and frequently managed in general practice, where non-pharmacological treatments are often used. General practitioners play a central role in the early identification and continued management of anxiety. Still, structural barriers such as brief consultation times and limited access to mental health resources can hinder the delivery of optimal care [19]. While a range of non-pharmacological interventions has become available, these vary widely in type and delivery. Most existing reviews have focused on specialist psychiatric settings or single intervention types, resulting in limited and inconsistent evidence available to guide in a general practice setting [18]. This underscores the need for a comprehensive synthesis of non-pharmacological options applicable to general practice. To address this, a scoping review was chosen to capture the range of available evidence.

In this review, general practice is understood as the organizational setting of the GP clinics itself. This includes interventions that are initiated, delivered or closely supported by GPs or personnel embedded within the practice. Interventions primarily relying on referral to external psychological, municipal or specialist services, even when accessed via primary care, were considered outside the scope of this review. The review therefore focuses on non-pharmacological interventions targeting patients with mild to moderate anxiety at an early stage, where treatment may be initiated within general practice before referral becomes necessary.

### Aim

This scoping review aims to explore and describe existing evidence on non-pharmacological interventions for anxiety in general practice, providing an overview of available treatment approaches and identifying gaps to inform future research.

## Method

We conducted this scoping review following the PRISMA Extension for Scoping Reviews (PRISMA-ScR) [20]. The PRISMA-ScR guidelines are designed to improve the transparency and accuracy of scoping reviews by providing a standardized framework for reporting. The guidelines include key elements such as a relevant title and abstract to identify the review’s scope, followed by an introduction outlining the research question. The methodology section details the search strategy, inclusion and exclusion criteria. In the ‘Results’ section, the study selection process is illustrated using a flow diagram, along with a summary of the included studies and their key findings. Finally, the discussion interprets the results, addresses the limitations of the review, and considers the literature’s implications.

### Search strategy

We completed a systematic literature search in three scientific databases: PubMed, APA PsycInfo and CINAHL. We developed the search strategy based on the PICO framework [21] to identify relevant studies on non-pharmacological treatments for anxiety in general practice. A research librarian from the University of Copenhagen assisted in the search process, ensuring a broad and structured approach. In databases that support MeSH (Medical Subject Headings) terms, such as PubMed, we used MeSH terms for more search precision. Specifically, the search included terms like *‘Anxiety Disorders’[MeSH]* to identify studies related to anxiety conditions, and *‘Psychotherapy’[MeSH]* to cover various non-pharmacological interventions. To ensure a broader search, MeSH terms were combined with free-text searches. Boolean operators (AND/OR) were used to further refine the search. See the supplementary file for search strategies from all databases. The searches were run on 11 February 2025, and limited to peer-reviewed studies published in English between 2015 and 2025. Limiting the inclusion of literature to studies published within this time frame ensures that the review is based on the current and clinically relevant evidence. Grey literature, such as unpublished reports, was excluded to maintain consistency in methodological quality and reporting standards.

### The PICO criteria

The following PICO framework was used to guide the search.

*Population*: The population of interest included adults aged 19 years and older diagnosed with either generalized anxiety disorder (GAD), obsessive-compulsive disorder (OCD), social phobia, panic disorder or specific phobias. Studies were included if they were conducted in countries with a general practice healthcare system as the primary access to health care, to ensure the findings are relevant and applicable to countries with similar systems.

*Intervention*: Non-pharmacological treatments that are applicable in general practice and delivered by personnel trained to support the intervention within the general practice framework. It has to be a therapeutic intervention that takes place in a clinical context and without referral to another health care professional. This also means that group interventions have been excluded. Studies were also excluded if the interventions were delivered primarily through external primary care, municipal or specialist services accessed via referral, even if patients were recruited from general practice.

*Comparison*: The comparator component was not specified as an eligibility criterion. As this was a scoping review, studies with or without comparators were eligible, as the aim was to map intervention characteristics rather than to assess comparative effectiveness.

*Outcome*: Reduced anxiety.

### Study selection

**Figure 1.**
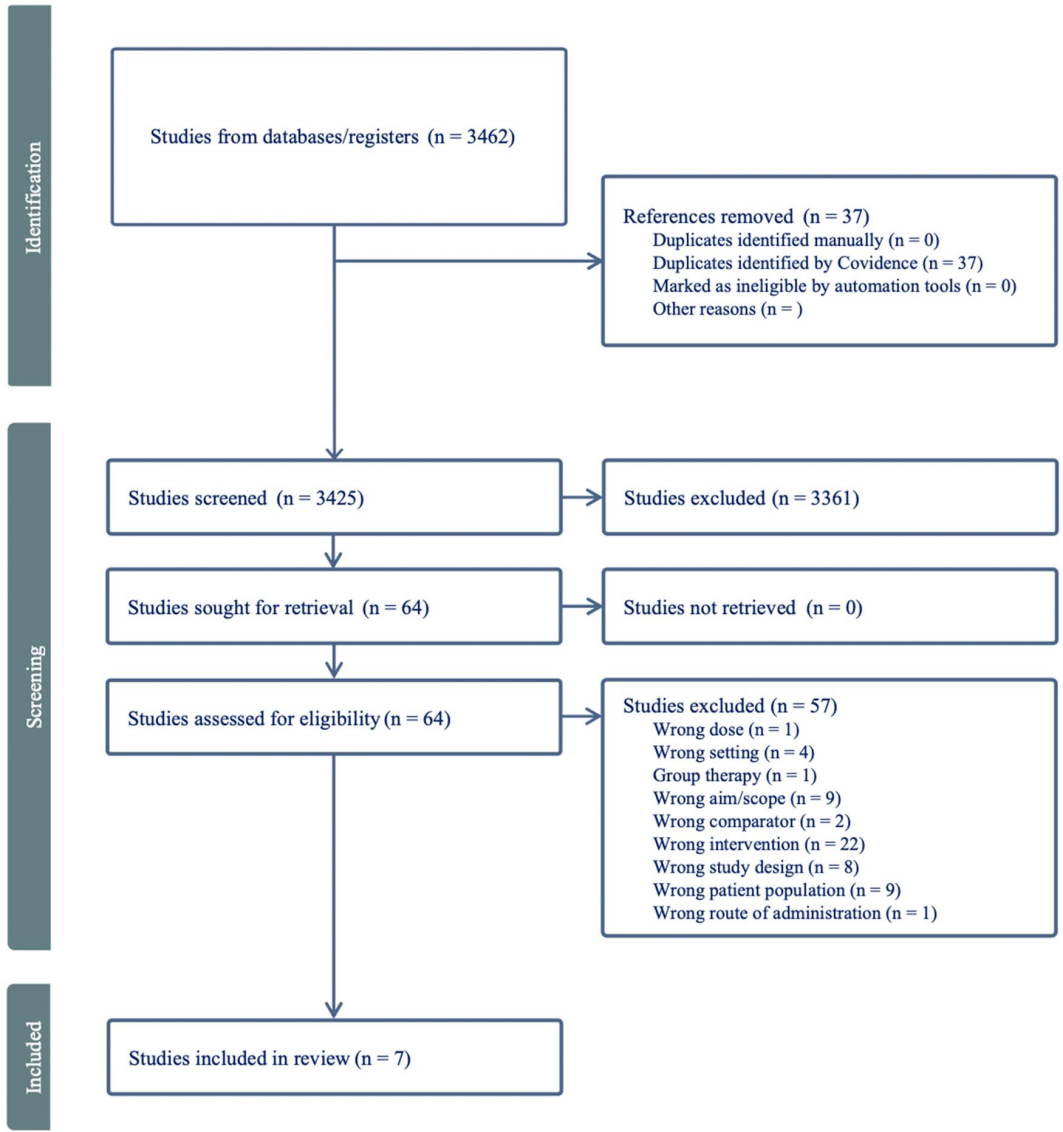
PRISMA-ScR flowchart presenting the study selection process in the systematic scoping review.

Authors independently screened using Covidence [22], a software tool designed to facilitate reviews. The screening process is depicted in the flowchart in Figure 1. First, studies were assessed based on title and abstract. Potentially relevant studies were then reviewed in full text to determine final inclusion. In addition to CBT-based approaches, the included studies examined interventions such as ACT, body-based interventions and low-intensity stepped-care models. Interventions such as mindfulness and psychoeducation were eligible if delivered within the general practice setting but were not identified in the included studies. Covidence allowed for efficient collaboration between my co-author and me and ensured that all studies were strictly evaluated. Any disagreements between us were resolved through discussion.

### Data extraction and assessment

Data from the included studies were extracted systematically using the PRISMA extension checklist for scoping reviews [23]. Extracted information included: author, year of publication, country, study objectives, study design, number of participants, description of participants, who delivers the intervention, description of participants, mode of delivery, the intervention and main results. Data were collected and presented in Tables 1 and 2.

**Table 1.** Characteristics of the included studies investigating non-pharmacological interventions for anxiety in general practice.

First author with reference	Year of publication	Country	Study objectives	Study design	Number of participants
De Lorent et al. [24]	2016	Germany	Comparison of the effect of auricular acupuncture with progressive muscle relaxation in patients with anxiety disorders and/or depression.	Prospective, parallel group clinical trial.	162
Witlox et al. [25]	2021	The Netherlands	Investigate the effectiveness of a mixed acceptance commitment therapy (ACT) intervention for adults with anxiety symptoms compared to face-to-face cognitive behavior therapy (CBT).	Pragmatic, single-blind cluster randomized trial.	314
Graham et al. [26]	2020	U.S.A.	Digital intervention via app with coaching support. Evaluating the effectiveness of a mobile intervention platform, IntelliCare, in addressing depression and anxiety among general practice patients.	Two arm randomized clinical trial	146
Chermahini et al. [27]	2024	Canada	Investigation of the effects of weekly online check-ins and compare it to a group receiving online cognitive behavior therapy.	Randomized controlled trial	96
Salomonsson et al. [28]	2018	Sweden	Stepped care model for common mental disorders in general practice.	Randomized controlled trial	396
Lukaschek et al. [29]	2019	Germany	Identification and characterization of symptom trajectories in general practice patients with panic disorder with/without agoraphobia who participated in a general practice team-based training involving cognitive behavior therapy.	Secondary analysis from a randomized controlled trial	176
Newby et al. [30]	2017	Australia	A comparison between the effect of transdiagnostic I-CBT with disorder specific I-CBT in general practice.	Non-randomized controlled trial	2109

The table summarizes study objectives, design, country and sample size for each included study.

**Table 2.** Mode of delivery, setting and main findings from the included studies.

First author with reference	Who delivers the intervention	Duration	Description of participants	Mode of delivery	The intervention	Anxiety assessment method	Main results
de Lorent et al. [24]	Healthcare staff trained in acupuncture and progressive muscle relaxation (likely nurses, physiotherapists or psychologists).	Twice a week over four weeks.	Adults with primary diagnosis of anxiety disorder or depression.^a^	In a clinical setting.	The received either auricular acupuncture (AA) or progressive muscle relaxation (PMR).	VAS	Both interventions significantly reduced anxiety (AA: 49.0 → 35.0; PMR: 47.8 → 34.6; *p* < 0.001 in both), with no significant difference between groups (*p* = 0.85).
Witlox et al. [25]	Mental health counsellors Psychologists = 13 Social psychiatric nurses = 14 Social workers = 5 System therapist = 2 Different educational background *n* = 6	One session a week in 8 weeks. Follow-up after 6 and 12 months.	Adults age 55–75 with mild to moderate anxiety symptoms.	In a general practice and the intervention was a mixed between online and in-person sessions with a mental health professional.	Participants were randomized to either ACT combining online modules with in-person sessions compared to standard face-to-face CBT in general practice.	GAD-7	Both blended ACT and face-to-face CBT significantly reduced anxiety symptoms, with no significant difference between treatments. ACT was associated with better long-term mental health outcomes and higher treatment satisfaction (Cohen’s *d* = 0.96 for ACT; *d* = 1.09 for CBT).
Graham et al. [26]	Coaches were two bachelor’s degree level individuals who received training in the coaching manual and weekly supervision with a clinical psychologist.	8 weeks, with participants being encouraged to use app regularly throughout.	Adults general practice patients who screened positive for anxiety or depression.^a^	The intervention was delivered online via app’s supported by text-based coaching.	Participants engaged with a mobile app intervention supported by text-based coaching.	GAD-7.	A greater proportion of intervention compared to waiting list control participants achieved significant recovery from depression and anxiety. The intervention group had 2.17 higher odds for recovery from anxiety.
Chermahini et al. [27]	Licensed mental health professionals, likely psychologists.	8 week intervention with asynchronous feedback or weekly check-ins.	Patients diagnosed with general anxiety disorder.	Online intervention with asynchronous feedback from therapist.	Randomization to either online therapy program or a program with weekly check-ins. Both groups received asynchronous digital communication	GAD-7.	Both treatments demonstrated statistically significant reductions in GAD-7 scores, but there was no significant different between the treatments.
Salomonsson et al. [28]	14 licensed psychologists.	Guided self-help for 10 weeks, additional 10 weeks of face-2-face for non-responders.	General practice patients with anxiety, depression, adjustment or exhaustion disorder.	Conducted in a general practice and step I was low intensity guided and step II with more sessions.	Participants first received guided self-help CBT. Non-responders randomized to face-to-face CBT or continued self-help.	Liebowitz Social Anxiety Scale-Self report.	After step I, 40% patients were in remission. After step II, 39% of patients following face-to-face CBT were in remission compared with 19% of patients after continued GSH.
Lukaschek et al [29]	Doctors or nurses trained in CBT principle.	Four to six face-to-face sessions over a 12-week period, supported by telephone contacts.	General practice patients with panic disorder with/without agoraphobia.	Intervention was in a general practice setting with a follow-up by telephone.	CBT was delivered by trained general practice teams, including face-to-face sessions and telephone follow-ups.	OASIS scale.	Most participants (83.5%) responded positively to the intervention, showing sustained reductions in anxiety symptoms.
Newby et al. [30]	General practitioner provided support to the online intervention.	8-lesson online course completed over approximately 10 weeks, with general practitioner encouragement	Patients in general practice with anxiety and depression.	Online intervention with in person encouragements from general practitioner.	Participants completed a transdiagnostic or disorder-specific I-CBT program over 10 weeks with GP support.	GAD-7	Both transdiagnostic and disorder specific I-CBT are effective in general practice with small alterations favorizing transdiagnostic I-CBT with an effect size *d* = 0.44).

The table outlines the type of intervention, delivery format and key outcomes related to anxiety symptom reduction.

^a^ Meaning some of the participants were in pharmacological treatment.

Finally, to support clarity and language quality, grammatical refinements and minor language adjustments were assisted by ChatGPT (OpenAI). All content, interpretation and academic judgment remain the responsibility of the authors.

## Results

### Study selection

A total of 3462 articles were identified. After removing 37 duplicates, 3425 titles and abstracts were screened. Sixty-four full-text studies were assessed for eligibility, and seven studies were included for review.

### Study characteristics

Table 1 presents the characteristics of the seven studies included. They were published between 2016 and 2024 and varied in design and intervention type. Five were randomized controlled trials, and two were observational or effectiveness studies. The studies were carried out across various healthcare systems, including those in the Netherlands, Germany, Ireland, Australia, Sweden, Canada and the United States. All studies were conducted in or closely associated with general practice settings. The studies primarily targeted adults with anxiety disorders, particularly GAD. Some studies also included adults with depression [25–28,30]. The included studies except one, clearly differentiated between outcomes for anxiety and depression in their results [28].

Digital and internet-based interventions were prominent in several studies, reflecting the growing interest in technology-enhanced treatments [31]. The study sample sizes ranged from moderate to large, typically between 100 and 300 participants. The outcomes were assessed using anxiety scales such as GAD-7, OASIS and VAS.

### Quality assessment

Across the seven studies, most provided clear inclusion and exclusion criteria, with well-described aims and target populations. The interventions and control conditions were generally described in sufficient detail to allow for replication, although a few studies lacked full transparency about therapist qualification [26,27]. Validated outcome measures, such as the GAD-7, OASIS and PHQ-9, were widely used, strengthening the anxiety assessments. Dropout rates were inconsistently reported, with two studies [24,26] lacking exact numbers or the reason for completion. The statistical methods used were appropriate for the study designs and included techniques such as mixed models, logistic regression and growth mixture modelling.

### Synthesis of results

Across the seven studies included in this review, three overall categories of non-pharmacological interventions for anxiety in general practice were identified. Four studies examined digital and technology-assisted interventions [25–27,30], while one study focused on a physical and body-based intervention [24]. The remaining two studies evaluated step-wise or low-intensity care models embedded in general practice settings [28,29]. The interventions varied in format and provider involvement, highlighting the variety of treatment options for anxiety in general practice. Tables 1 and 2 show an overview of the characteristics of the included studies.

### Digital and technology-assisted interventions

Digital and technology-assisted treatments for anxiety in general practice were explored through a range of interventions, including mobile app-based platforms, internet-delivered cognitive behavioral therapy (I-CBT) and blended formats that combined online modules with in-person consultations.

Several interventions using digital or blended formats were explored for the treatment of anxiety in general practice settings. In one study [26], a collection of self-guided mobile applications, available on iPhone and Android, supported by text-based coaching, was evaluated over 8 weeks. Participants received onboarding support and optional calls during and after the intervention. The apps were self-guided, and the authors describe them as being of low intensity to make them minimally resource-demanding. Recovery rates were significantly higher in the intervention group (57%) compared to a waitlist control group (38%), with 2.17 times greater odds of recovery from anxiety. Mean GAD-7 scores declined steadily from 11.6 at baseline to 8.4 at week 4 and 6.8 at week 8, with effects maintained at a 16-week follow-up.

A comparison between structured I-CBT and weekly online health check-ups examined how different methods of therapeutic support may influence outcomes [27]. Participants were recruited through primary care settings and referred to one of the two low-intensity digital treatment arms. Participants in the I-CBT group completed structured modules with asynchronous therapist feedback. This allowed patients to work at their own pace while still receiving professional guidance. The second group engaged in weekly digital check-ins, where they responded to structured prompts without receiving formal CBT-based content. All communication in both groups was asynchronous, offering a flexible and minimally resource-intensive intervention format suited to a general practice population. About 46% of participants in the I-CBT and 45% in the check-in group completed the 12-week program.

Anxiety symptoms were measured using the GAD-7 scale. Both groups showed statistically significant reductions in GAD-7 scores (I-CBT: from 13.18 to 9.88; check-in: from 13.55 to 10.44), but no significant difference was found between the groups (*F* = 0.024, *p* = 0.878) (Table 2). These findings suggest that structured digital communication alone may be sufficient to reduce anxiety symptoms in some patients.

A large real-world implementation study in Australia compared transdiagnostic I-CBT to disorder-specific I-CBT for treatment of GAD and depression in a large general practice sample [30]. The patients were referred by staff from primary care to the two types of I-CBT. The online platform delivered six structured self-guided lessons. The supervising clinicians were encouraged to contact the patients twice during the program, and they were notified if the patients’ distress levels increased. The completion rates were 44.9% for the transdiagnostic program and 49.2% for the GAD program. All the intervention types showed significant improvements in anxiety symptoms. The transdiagnostic program showed small but statistically significant advantages over disorder-specific I-CBT in reducing GAD-7 scores (*d* = 0.48, 95% CI: 0.36–0.60), especially in patients with comorbid depression.

In the Netherlands, a pragmatic cluster-randomized trial [25] compared a partly online blended ACT with traditional face-to-face CBT in older adults aged 55–75 years with mild to moderate anxiety symptoms in general practice. The interventions were delivered by health counselors integrated into general practice. Both interventions led to significant reductions in anxiety symptoms, with large effect sizes (Cohen’s *d* ≥ 0.96), and these improvements were maintained at 12-month follow-up. No significant differences were found between groups regarding anxiety outcomes. However, treatment satisfaction was significantly higher in the ACT group compared to CBT, and participants in the ACT group also reported improved mental health scores at follow-up. These findings suggest that blended ACT may be a valuable alternative to traditional CBT in older populations, particularly because its digital format offers greater flexibility and may reduce barriers related to stigma associated with seeking mental health treatment.

### Physical and body-based intervention

Auricular acupuncture and progressive muscle relaxation were tested as non-pharmacological treatments for GAD over four weeks in a clinical setting [24]. Participants freely chose between the two interventions and received guidance from certified clinical staff. Both treatments led to significant reductions in anxiety scores (auricular acupuncture: from 49.0 to 35.0; progressive muscle relaxation: from 47.8 to 34.6; *p* < 0.001 for both). No significant difference was found between the two groups (*p* = 0.85). The study does not provide exact dropout rates, but the authors explicitly mention that dropout was an issue, especially in the PMR group.

### Step-wise and low-intensity care models

These studies examined a needs-based approach to care, typically starting with low-intensity interventions and increasing in intensity only when necessary. Two studies in this review explored such stepped care frameworks, which aim to improve treatment accessibility and efficiency by minimizing therapeutic involvement at the outset and reserving more intensive resources for those who require them [28,29].

A stepped care model was implemented in Swedish general practice clinics, using guided self-help CBT as the initial intervention [28]. Participants received brief support from trained therapists during step I, with therapist involvement increasing for those who progressed to step II. After 9 weeks of intervention, 40% of the patients were in remission. The rest were randomized to either continue guided self-help CBT or face-to-face CBT, where face-to-face CBT showed a quicker effect (remission: 39% versus 19%). In step II, 161 out of 214 non-responders agreed to randomization. At the 6- and 12-month follow-up, there was no significant difference between the groups. The authors concluded that face-to-face CBT provided an additional benefit for those who did not respond to guided self-help, leading to faster remission.

In Germany, the intervention was implemented in routine general practice settings, where both GPs and medical assistants were trained to provide structured mental health support. Patients received psychoeducation and CBT-based exposure exercises delivered by the GP. They also received a printed workbook for home use along with regular telephone follow-up by medical assistants. The GP conducted four structured sessions, and the medical assistants did the telephone follow-up using a standardized checklist, while encouraging. The purpose of the medical assistants is to do a follow-up and lighten the burden on the GPs. A ‘Grow mixture modeling’ analysis identified three course profiles: 33% (*n* = 58) had gradual improvement, 51% (*n* = 89) had rapid improvement and 16.5% (*n* = 29) had an unstable course with limited improvement.

## Discussion

### Summary of main findings

This scoping review identified only seven relevant studies examining non-pharmacological interventions for anxiety in general practice, suggesting that research in this area remains limited. It is striking how little literature specifically addresses non-pharmacological approaches adapted to the realities of general practice, despite the high prevalence of anxiety disorders and the central role of GPs in their management.

We identified three main categories of non-pharmacological interventions for anxiety delivered within a general medical practice setting: digital and technology-assisted treatments, stepwise and low-intensity care models, and a body-based intervention. The majority of interventions were designed to be feasible in a general practice setting, requiring minimal specialist resources or referral to secondary care. Several studies reported significant reductions in anxiety symptoms across different formats, including mobile apps, guided self-help CBT and acupuncture treatment. Digital interventions and stepped care models appeared particularly well-suited for integration into general practice due to their flexibility. However, heterogeneity in intervention designs, follow-up periods and outcome measures complicates direct comparison across studies.

### Relating to scientific literature

Our findings highlight the central role of GPs in initiating and supporting non-pharmacological treatment for anxiety. This resonates with previous research emphasizing the importance of continuity and therapeutic alliance in primary care. Hansen et al. recently synthesized the literature on relational competence in general practice, showing that trust, openness and continued connectedness are crucial attitudes and actions underpinning effective GP–patient interactions. Such relational competence can be seen as a prerequisite for successful management of mental health problems in primary care [32].

In practice, this means that when GPs build on their existing knowledge of the patient and foster these relational qualities, they are better positioned to initiate timely interventions, reduce unnecessary delays and provide treatments that patients are more likely to accept and adhere to.

In addition to relational factors, a growing body of evidence points to the potential of digital tools, physical activity and complementary approaches as part of anxiety management in primary care. Digital solutions, including remote consultations and app-based interventions, are valued by both GPs and patients for their accessibility and flexibility, and app-based programs have shown effectiveness for depression even without therapist involvement [33–35]. Mobile apps may therefore serve as a low-intensity, first-step option in stepped care models, allowing patients to trial self-management before escalation. Stepped care itself has been shown to improve both symptom response and remission while ensuring resource-efficient treatment [36]. Similarly, physical activity has demonstrated benefits: even minimal exercise is linked to reduced anxiety symptoms, and structured interventions in primary care have improved quality of life and work ability [37,38]. While these interventions may require collaboration with physiotherapists, they highlight the feasibility of embedding lifestyle-based approaches in general practice. Finally, the inclusion of acupuncture and other complementary modalities in this review reflects a growing interest in body-oriented strategies. Nearly, half of Scandinavian adults report using complementary and alternative medicine, particularly those with frequent GP contact [39], and evidence suggests that yoga and similar approaches can reduce anxiety symptoms [40]. Together, these findings indicate that general practice can play a central role not only through relational competence but also by integrating digital, lifestyle and complementary approaches into stepped care pathways for anxiety management.

## Summary and clinical implications

Taken together, the literature suggests that GPs are uniquely positioned to deliver early, non-pharmacological interventions for anxiety by combining strong relational competence with accessible treatment options. Digital tools and stepped care models can enable timely and resource-efficient support, while physical activity and complementary approaches provide additional low-intensity pathways that patients may find acceptable. Strengthening relational skills, providing access to digital solutions, and building collaborations with allied health professionals could therefore enhance the role of general practice in managing anxiety and help address the gap created by long specialist waiting times.

## Strengths and limitations

This review demonstrates an inconsistent reporting of participant dropout across the included studies. While some studies reported dropout rates and applied intention-to-treat analyses to mitigate bias [25,27,28,30], others provided limited or incomplete information on attrition [24,26,29]. This lack of transparency makes it difficult to fully assess treatment adherence and may lead to an overestimation of intervention effectiveness. Insufficient reporting on reasons for dropout restricts the ability to evaluate the interventions in routine general practice settings. Future studies should report how many participants dropped out and why they dropped out, to evaluate effectiveness in a general practice setting.

In several studies, participants were self-selected by responding to invitations or referred by their GPs [26,28,29], which may have introduced selection bias favoring individuals who were already motivated to engage in non-pharmacological treatments [41].

Given the concerns around long-term pharmacological treatment, especially in elderly patients, broader non-pharmacological approaches are needed. Benzodiazepines are often prescribed in large quantities despite guidelines recommending short-term use [42,43]. Prolonged use of addictive medications is associated with risks such as tolerance, cognitive decline and increased fall risk, while non-pharmacological alternatives can offer equal or greater effectiveness [44,45].

Five of the studies included patients with comorbid conditions such as depression [24–26,28,30] or panic disorder [29], further contributing to heterogeneity in patient characteristics. While this diversity can complicate the interpretation of intervention effects for isolated anxiety disorder, it may also enhance the clinical relevance of the findings. Comorbidity is common in general practice, and patients with overlapping symptoms, particularly those with unipolar depression secondary to untreated anxiety, are frequently encountered [46,47]. Although comorbid severe mental disorders, such as bipolar disease, were excluded, we did not exclude studies with patients with unipolar depression to reflect the reality of general practice and support the applicability of the interventions to the population within.

Two studies allowed participants to continue pharmacological treatment for anxiety during the intervention period [25,29], and it could have influenced symptom improvements independently of the non-pharmacological interventions. However, because many patients in general practice have other health problems and are already taking medication, the study reflects real-world conditions well.

Several internationally established primary-care psychological treatment systems, including IAPT in the UK, national internet-delivered CBT (iCBT) platforms in Denmark, and Internetpsykiatri in Sweden, provide evidence and the feasibility for non-pharmacological treatment of anxiety disorders [48–50]. These systems typically deliver CBT-based interventions via referral-based services external to the GP practice. While highly relevant at a primary-care level, they differ organizationally from interventions delivered within or embedded in general practice and were therefore not included in the present review.

The focus on interventions delivered within the organizational setting of general practice may have limited the number of eligible studies. This reflects a deliberate methodological choice and should be considered when interpreting the findings. By focusing on intervention delivery and organizational context, this review complements the existing effectiveness literature by highlighting how and where non-pharmacological anxiety treatments are implemented within general practice, and where evidence remains limited.

Although CBT and iCBT are among the most extensively studied non-pharmacological treatments for anxiety, much of this evidence originates from referral-based primary-care services rather than from decentralized, co-located general practice teams. There is a notable lack of studies examining anxiety interventions delivered within general practice settings where GPs and other health professionals work side by side. This organizational gap may partly explain why evidence on practice-embedded non-pharmacological anxiety treatment remains limited despite a large overall literature base.

A substantial body of evidence supports the effectiveness of CBT and iCBT for mild to moderate anxiety disorders at primary-care level, as demonstrated in meta-analyses, community-based trials and routine-care evaluations, and reflected in international clinical guidelines. The present review does not aim to reassess this evidence base, but rather to examine how non-pharmacological interventions, including CBT-based approaches, have been delivered within the organizational setting of general practice.

### Implications for clinical practice

Although only a few studies have explored non-pharmacological treatments for anxiety in general practice, existing evidence suggests that such interventions are both feasible and effective. Given the high prevalence of anxiety among patients and the limited access to specialized mental health care services, many patients naturally seek treatment in primary care. Due to the GP’s central role and ongoing patient contact, initiating and monitoring non-pharmacological treatments should be relatively easy, allowing for continuous reassessment of the need for pharmacological intervention as well as differential diagnoses, while avoiding unnecessary referrals that may increase the risk of pathologizing the patient. GPs could consider integrating self-help strategies, digital tools, or complementary and alternative medicine into their treatment options. However, caution is warranted in more complex cases or when severe comorbidities are present, where referral to specialized care remains necessary.

### Implications for research

The current research on non-pharmacological treatment of anxiety in general practice is sparse. There is a need for more studies that assess clinical effect and practicability. Research should also focus on identifying which interventions are most acceptable and useful for diverse patient groups, including those with common comorbidities such as depression. This scoping review highlights the need for further investigation into non-pharmacological treatment possibilities within the general practice setting.

## Conclusions

This scoping review highlights that while the evidence is still limited, a range of non-pharmacological interventions, such as digital tools, body-based interventions, low-intensity and stepped care, have shown promise in the treatment of anxiety in general practice. These interventions appear possible in real-world settings, particularly for patients with mild to moderate symptoms. An important knowledge gap identified by this review is the lack of studies examining non-pharmacological anxiety interventions delivered within decentralized, co-located general practice teams. Future research should focus on how such interventions can be integrated into routine general practice to support early, practice-based management of mild to moderate anxiety. Given the high prevalence of anxiety, there is a growing need for accessible, non-pharmacological solutions in general practice. Such approaches may offer accessible alternatives to traditional mental health care, making flexible, non-pharmacological strategies available as GPs manage more patients with anxiety and comorbidity, either as a supplement to or replacement for medication. To ensure these approaches can be effectively integrated into routine practice, more research is needed to explore their long-term effectiveness and implementation in diverse patient populations.
